# Epidemiology and outcomes of out of hospital cardiac arrest in Saudi Arabia: Findings from the Saudi Out of Hospital cardiac Arrest Registry (SOHAR)

**DOI:** 10.1016/j.resplu.2023.100516

**Published:** 2023-11-29

**Authors:** Abdullah Alabdali, Abdulrhman Alghamdi, Meshary Binhotan, Abdullah Alshibani, Meshal Alharbi, Alanowd Alghaith, Mohammad Altuwaijri, Saad Albaiz, Omar Aldibasi, Mohammed Alwarhi, Noura Alquraishi, Nawfal Aljerian

**Affiliations:** a Emergency Medical Services Department, College of Applied Medical Sciences, King Saud Bin Abdulaziz University for Health Sciences, Riyadh, Saudi Arabia; b King Abdullah International Medical Research Center, Riyadh, Saudi Arabia; c Medial Affairs Department, Saudi Red Crescent Authority, Riyadh, Saudi Arabia; d Department of Emergency Medicine, King Abdulaziz Medical City Riyadh, Ministry of National Guard Health Affairs, Riyadh, Saudi Arabia; e King Saud Bin Abdulaziz University for Health Sciences, Riyadh, Saudi Arabia; f Medical Referral Center, Ministry of Health, Riyadh, Saudi Arabia

**Keywords:** Emergency Medical services, Ambulance, Cardiac arrest, Registry, Epidemiology, CPR

## Abstract

**Aim:**

The Saudi Out-of-Hospital Cardiac Arrest Registry (SOHAR) is the first out-of-hospital cardiac arrest (OHCA) registry in Saudi Arabia. This study aimed to describe the epidemiology and outcomes of OHCA in Saudi Arabia.

**Methods:**

The SOHAR is a prospective data collection system. Data were collected monthly from defined regions, and registry measured variables were adopted from the Utstein recommendations.

**Results:**

During the period from 01/01/2019 to 31/12/2022, 3671 patients were included in the registry. The mean age was 62 years, and 6.5% (240) of patients were under the age of 18 years. The most common cause of OHCA was medical 3439 (93.6%). A total of 641 (17.4%) and 129 (3.9%) had presumed cardiac and respiratory causes. Additionally, most OHCA in Saudi Arabia (3034, 82.6%) occurred at home. Prehospital Return Of Spontaneous Circulation (ROSC) was achieved in 275 (7.4%) cases, and 491 (13.3%) patients were pronounced dead upon arrival at the hospital. Survival to hospital discharge was achieved in 107 (2.9%) of the cases, and good neurological outcomes, defined as a Cerebral Performance Category (CPC) of 1–3, occurred in < 0.5% of patients.

**Conclusion:**

The Saudi out-of-hospital ROSC was 7.4%. The survival to hospital discharge rate was 2.9%, and less than 1% of patients were discharged with good neurological outcomes. Further research and the continuation of registry data collection is highly recommended. Additionally, a national-level out-of-hospital cardiac arrest system is recommended to ensure the standardization of medical care provided to patients with OHCA.

## Introduction

Out-of-hospital cardiac arrest (OHCA) is an international health issue, with a worldwide incidence of 55 cases per 100,000 individuals per year among adults.^1^ OHCA survival rates vary substantially by area, and OHCA has a poor prognosis without medical intervention. Thus, an early response to sudden cardiac arrest will result in a significant improvement in survival.^2^ The Gulf Cooperation Council (GCC) region has low survival rates, and data on OHCA are still lacking.^3^ Three studies in the United Arab Emirates, Saudi Arabia, and Kuwait have reported that the Return of Spontaneous Circulation (ROSC) in OHCA was 3.1%, 8.0%, and 3.5%, respectively. Previous research showed that Middle Eastern healthcare systems made no progress toward improving OHCA recognition, management, and outcome.^4^, ^5^, ^6^

Cardiac arrest registries have been established at regional and international levels. For example, the Pan-Asian Resuscitation Outcomes Study (PAROS) was founded in 2010 as a collaboration between countries throughout Asia.^7^ Another OHCA registry, developed in the United States, is the Resuscitation Outcomes Consortium Epistry-Cardiac Arrest,^8^ while Europe has established the European Registry of Cardiac Arrest (EuReCa).^9^ More registries have been established worldwide to help understand the variation in OHCA cases.

The Saudi Out-of-Hospital Cardiac Arrest Registry (SOHAR) is the first and only out-of-hospital cardiac arrest registry in Saudi Arabia. It was established in January 2019, and every incidence of OHCA attended to by local Emergency Medical Services (EMS) through the Saudi Red Crescent Authority (SRCA) has been recorded in the secured SOHAR database.^10^ SOHAR follows the Utstein style, which is an internationally recognized guideline for reporting OHCA.^11^ SOHAR provides useful and reliable information that can be used to develop a good relationship between the variables affecting OHCA survival in Saudi Arabia. This study aimed to describe the epidemiology and outcomes of OHCA in Saudi Arabia.

## Materials and methods

### The EMS system

SRCA is the sole provider of emergency medical services (EMS) for the vast majority of the country. It offers prehospital care through emergency calls and disaster response. The SRCA operates with qualified emergency medical technicians (EMTs), paramedics, and doctors; it is mainly funded and supported by the government. The estimated total number of SRCA clinical providers is more than 7000. Emergency calls are received via regional central dispatch centers with more than 100,000 calls per month.

### The registry

The registry is a prospective data-collection system, and data were collected monthly from the selected regions. Saudi Arabia is subdivided into 13 regions (provinces) including 40 cities, 20 of which are considered major cities. Each city had a designated registry coordinator responsible for data collection and entry. Data were entered into an online database designed to fulfil the registry requirements, and the registry included all OHCA events starting from 01/01/2019 that were reported from the Saudi EMS provider. The registry is covering Saudi citizens and residents requesting EMS services in included regions. The registry is funded by King Abdullah International Medical Research Centre (KAIMRC) therefore the registry is operating under the supervision of KAIMRC with quarterly progress reports provided by the registry primary investigator. The registry hired project manager, registry coordinators for each city, individual data collectors and support staff such as office assistants. The administration operation is under the project manager and scientific operations are handled by the registry investigators.

Ethical approval was obtained from KAIMRC (RC20/154/R).

### Data collection

The patient data were collected from two main sources:

• The Saudi EMS provider, the SRCA
• The Saudi receiving hospitals

The reported cases of OHCA from the SRCA were followed up by the research support team, and each patient was assigned a unique identification number using an online system (REDCap). All patients were followed-up to record their hospital outcomes. The registry has a unified form of reporting OHCA, using the 2015 update of “Utstein.” The following core datasets were included:

• System core
• Dispatch core
• Patient core
• Process core

An online registry with limited access, granted by the project director, was available to city coordinators; coordinators alone were able to enter data. Each case entry was assigned a unique serial number based on the city. Incomplete data were not issued a number requiring review and re-submission from the coordinator.

### Inclusion and exclusion criteria

All patients with OHCA transported to a hospital by the Saudi Red Crescent ambulances were included. The exclusion criteria were: (1) patients announced dead on the scene; and (2) patients with a “do-not-resuscitate” order, with no attempt of resuscitation at the time of arrival at the hospital.

### Outcomes

The registry measured variables adopted from the Utstein recommendations.^11^ First, demographic information such as date of birth, sex, and nationality were included. Second, detailed information was collected regarding the prehospital phase, including the method of transport, the time the EMS received the call, and the time the ambulance was dispatched, arrived, left the scene, and arrived at the emergency room (ER). The prehospital data included the patient’s past medical history, the location of the incident, the estimated time of arrest, the first arrest rhythm, the time of cardiopulmonary resuscitation, the time the automated external defibrillator was applied, airway intervention, medication administration, and details of the resuscitation process. The third phase contained detailed information on the patient’s status in the ER, including interventions. The last phase was patient survival outcome, including the Cerebral Performance Category (CPC).

### Statistical analysis

We used SAS 9.4 (SAS Institute, Cary NC) to perform exploratory and descriptive analyses on the registry data. First, we examined the data for outliers. We then reviewed and cleaned the data to address any issues we found. Finally, we performed descriptive statistics to summarize the registry variables, including patient demographics, response time in minutes, and outcome variables.

## Results

During the period from 01/01/2019 to 31/12/2022, a total of 3671 patients were included in the registry; 2296 (62.5%) were male and 1375 (37.5%) were female. The incidence of EMS attended OHCA is 23 per 100,000 people. The median (Q1,Q3) age was 60 (43,75) and 240 (6.5%) patients were <18 years old. The majority of patients (2514, 68.5%) were Saudi citizens. Table 1 presents the baseline patient characteristics.

**Table 1:** Characteristics of SOHAR patients.

Characteristic (*n* = 3671)	Value
Resuscitation attempt by EMS* (%)	3662 (99.8)
Median age in years (age range)	62 (0–109)
Sex, *n* (%)	
Male	2296 (62.5)
Female	1375 (37.5)
Pediatrics < 18 years (%)	240 (6.5)
Cause of Arrest	
Medical (%)	3439 (93.6)
Trauma (%)	232 (6.4)
Presumed cause of medical arrest (*n* = 3439)	
Cardiac (%)	641 (18.6)
Respiratory (%)	129 (3.8)
Saudi nationals (%)	2514 (68.5)

The median (Q1,Q3) response time from call to arrival in minutes was 13 (9,18). The median scene time was 10 (7,16).

The most common cause of OHCA was medical 3439 (93.6%). A total of 641 (17.4%) and 129 (3.9%) had presumed cardiac and respiratory causes Most patients (2699, 73.5%) had past medical history recorded. The most common pre-existing conditions were diabetes (1341, 36.5%), hypertension (1178, 32.9%), and history of heart disease (838, 22.8%).

The locations of OHCA are summarized in Table 2. Most OHCA cardiac arrests in Saudi Arabia occurred at home 3034 (82.6%); the exact location coordinates of cardiac arrests are available for 81% of the cases in our registry.

**Table 2:** Location of prehospital cardiac arrest.

Location (*n* = 3671)	Frequency (%)
House resident	3034 (82.6)
Street	335 (9.1)
Public/Commercial building	96 (2.6)
Health care facility	48 (1.3)
Recreational centre	21 (0.6)
Brought to ambulance station	16 (0.4)
School	7 (0.1)
Ambulance	6 (0.1)
Other	108 (2.9)

The estimated time of arrest was available for 2764 (75%) cases, and 1757 (47.8%) cases were witnessed. The majority of arrests were witnessed by family members (1101, 29.9%), while 656 (17.8%) were witnessed by bystanders. Dispatch-assisted CPR was instructed by dispatcher in 2375 (64.6%), respectively. The AED was applied by bystander in 53 (1.4%) of the cases. First CPR was initiated in most cases by ambulance staff (2384, 64.9%), bystanders (1017, 27.7%). The ambulance staff attempted to resuscitation in 3655 (99.6%) cases. The first cardiac arrest rhythm in 2370 (64.5%), 382 (10.4%), 191 (5.2%), and 728 (19.8%) cases was asystole ventricular fibrillation/ventricular tachycardia/shockable rhythm, pulseless electrical activity, and not recorded/not applied, respectively. Prehospital defibrillation was performed in 567 (15.5%) cases. The initial airway management was manual airway management (manual hand maneuvers) in 1207 (32.8%), basic (oropharyngeal airway or nasopharyngeal airway) in 928 (25.2%), advanced (supraglottic devices and endotracheal tube) in 304 (8.2%), surgical in one, and not recorded in 1231 (33.5%) patients. Prehospital advanced life support medications and fluid therapy were administered to 325 (8.8%) patients. Table 3 shows the medications administered to patients.

**Table 3:** Advance life support medication and fluid administration.

Medication (*n* = 325 patients)	Frequency (%)
Normal Saline	325 (100)
Epinephrine	303 (93.2)
Amiodarone	8 (2.5)
Dextrose	4 (1.2)
Atropine	3 (1)
Sodium Bicarbonate	3 (1)

The prehospital ROSC was achieved in 275 (7.4%) cases and 491 (13.3%) patients were pronounced dead upon arrival at the hospital. Patient transferred with on-going CPR was 3328 (90.6%). Survival to hospital discharge was achieved in 107 (2.9%) cases, and good neurological outcomes, defined as a CPC of 1–2, occurred in <0.5% of patients.

## Discussion

In this prospective OHCA registry, the findings revealed that 7.4% of patients with OHCA achieved ROSC; however, <0.5% were discharged with good neurological outcomes. These findings are consistent with the The Gulf Cooperation Council (GCC) previously reported out-of-hospital ROSC.^12^ The low rate of ROSC and low incidence of OHCA in our study compared to that in published international data might be attributed to the low utilization of EMS in Saudi Arabia, as most patients in critical conditions are transported in private cars.^13^, ^14^ In addition, most patients presented late to EMS, as indicated by the estimated time of arrest.

This study showed that majority of cardiac arrests (82.6%) in Saudi Arabia occurred at home, with a higher incidence than previously reported.^13^ The location of the arrest is important in determining future OHCA improvement targets.

Dispatch-assisted CPR was initiated in 64.4% of the cases; however, in the majority of cases, CPR initiation was through ambulance staff (64.9%). Theoretically, public CPR campaigns and teaching CPR to non-healthcare providers will have a significant impact on initiating CPR earlier, which may improve the ROSC rate. Further investigation is required to explore the low rate of bystander CPR initiation, despite the high rate of dispatch-assisted CPR. It is known that Saudi Arabia lacks public access to AEDs, and our data suggest that public access to AEDs must be carefully ensured in society. Public-access AEDs must be within the reach of local residents; mosques might be a good location to place public-access AEDs. In most cases, the first cardiac arrest rhythm was asystole/non-shockable rhythm. This finding might be attributed to the late presentation or the fact that this was the initial cause of arrest, which cannot be explained until further investigation. The late presentation to the EMS may be due to a lack of public awareness of the Saudi EMS phone number.^15^ There is a government initiative to unify public services using one unified phone number. This has already been implemented in a few regions and further analysis of the initiative is needed to determine its efficacy.

The Saudi EMS system is diverse with different levels of providers; the crew attending out-of-hospital cardiac arrest cases might be emergency medical technicians (EMT) with basic training, paramedics, or occasionally, physicians. This variation limits the ability to precisely determine the first initial rhythm because EMTs carry only AEDs, and cardiac monitors are only available to paramedics and physicians. Most of the cases in this study were attended by paramedics, and <20% were attended by EMTs.

The airway management in most patients (32.8%) was manual airway management involving manual maneuvers. This is a crucial finding in our registry. The assumption of maintaining the airway during the lengthy resuscitation process utilizing only manual airway management is questionable, and further training and protocol alterations must be implemented to ensure the efficacy of Saudi out-of-hospital resuscitation. Notably, 33.3% of the cases were missing the airway management data (not recorded). If we assume that there was no airway in these patients that should be considered a major deficit in the resuscitation process, and a system action is urgently required. If the EMS fails to document airway management, we recommend serious action to maintain adequate documentation.

The rate of prehospital ROSC in Saudi Arabia was 7.4%, which is comparable to the reported rate in our region.^12^ However, this rate is lower than the reported European and Asian ROSC rates of 36.7% and 22.1%, respectively.^1^ The low rate of Saudi prehospital ROSC requires further investigation and institutional collaboration to establish a prehospital cardiac arrest system in which patients with OHCA receive advanced medical care at an appropriate time. Our study showed that only 18.1% of patients were treated in a tertiary hospital. The discrepancies in the practice of resuscitation across the Kingdom of Saudi Arabia can be attributed to the size of the Kingdom and the differences in care providers between large and small communities. The establishment of an OHCA system in Saudi Arabia is highly recommended.^16^ The system should ensure standardization of medical care provided to patients with OHCA. Any recommended system must ensure that bystander CPR and public involvement in resuscitation are maximized. The SRCA has established a large public campaign to promote public CPR, however, other public entities and governmental institutions need to collaborate and promote public first aid, including the development of laws and legislation of public involvement.

Multiple difficulties were encountered in establishing an OHCA registry. First, the lack of collaboration between prehospital agencies and hospitals was the greatest barrier to obtaining data. The patient medical record and prehospital systems are two separate systems with no synchronization. The data obtained from the prehospital systems had to be followed-up manually and physically to ensure the completeness of data entry. This a synchronization eliminated 329 records from the registry. Second, the discrepancies in the hospital patient medical records system created a problem in obtaining hospital outcome data, as many hospitals did not have an electronic system, and we had to sort through papers to ensure that data were obtained. Finally, the unification of patient medical report numbers across all medical phases (prehospital, hospital, and rehabilitation centers) will have a significant impact on the collection of precise data.

## Conclusion

The Saudi out-of-hospital ROSC rate is 7.4%. Moreover, the survival to hospital discharge rate is 2.9%, and less than 1% of patients are discharged with good neurological outcomes. Further research and the continuation of registry data collection is highly recommended, and a national-level out-of-hospital cardiac arrest system is recommended to ensure the standardization of medical care provided to patients with OHCA.

## Funding

This work was supported by King Abdullah International Medical Research Centre (KAIMRC) [RYD-22-417780-191243, 2020].

## CRediT authorship contribution statement

**Abdullah Alabdali:** Conceptualization, Methodology, Validation, Formal analysis, Investigation, Data curation, Writing – original draft, Writing – review & editing, Supervision, Project administration, Funding acquisition. **Abdulrhman Alghamdi:** Conceptualization, Methodology, Validation, Data curation, Writing – review & editing, Project administration. **Meshary Binhotan:** Conceptualization, Methodology, Validation, Data curation, Writing – review & editing. **Abdullah Alshibani:** Conceptualization, Methodology, Validation, Data curation, Writing – review & editing. **Meshal Alharbi:** Conceptualization, Methodology, Validation, Data curation, Writing – review & editing. **Alanowd Alghaith:** Conceptualization, Methodology, Validation, Data curation, Writing – review & editing. **Mohammad Altuwaijri:** Conceptualization, Methodology, Validation, Data curation, Writing – review & editing, Conceptualization, Methodology, Validation, Formal analysis, Investigation, Data curation, Writing – original draft, Writing – review & editing, Funding acquisition. **Saad Albaiz:** Conceptualization, Methodology, Validation, Data curation, Writing – review & editing. **Omar Aldibasi:** . **Mohammed Alwarhi:** Conceptualization, Methodology, Validation, Data curation, Writing – review & editing. **Noura Alquraishi:** Conceptualization, Methodology, Validation, Data curation, Writing – review & editing. **Nawfal Aljerian:** Conceptualization, Methodology, Validation, Formal analysis, Investigation, Data curation, Writing – original draft, Writing – review & editing, Funding acquisition.

## Declaration of competing interest

The authors declare that they have no known competing financial interests or personal relationships that could have appeared to influence the work reported in this paper.

## Appendix A Supplementary material

Supplementary data 1 List of variables.
